# Autoimmune glial fibrillary acidic protein astrocytopathy presented as isolated area postrema symdrome: a case report

**DOI:** 10.1186/s12883-022-02802-2

**Published:** 2022-07-20

**Authors:** Jing Dang, Shengsuo Lei, Jihua Chen

**Affiliations:** The Department of Neurology, Chenzhou No.1 People’s Hospital, Chenzhou, Hunan 423000 People’s Republic of China

**Keywords:** GFAP, Encephalitis, Autoimmune, Hiccup, Area postrema symdrome

## Abstract

**Background:**

Area postrema syndrome (APS) as the isolated manifestation in autoimmune glial fibrillary acidic protein (GFAP) astrocytopathy has been rarely reported.

**Case presentation:**

A 61-year-old male patient presented with intractable hiccup. He was first admitted to the department of Gastroenterology because he had no symptoms other than hiccup. Then he was diagnosed with possible digestive system disease and started on treatment. 2 weeks later, his symptom didn’t improve at all. After consultation, the patient was referred to our department. Cerebrospinal fluid (CSF) analysis revealed lymphocytes pleocytosis, elevated protein level. Cell-based assays demonstrated GFAP antibodies in blood and CSF. His symptom improved with steroid pulse therapy (methylprednisolone, 1 g for 5 days), followed by a gradual tapering of oral prednisolone. Three months after the initial presentation, he showed no relapses.

**Conclusions:**

We report atypical manifestation of autoimmune GFAP astrocytopathy which presented as APS, suggesting that autoimmune GFAP astrocytopathy should be added to the list of possible cause of APS.

## Background

Autoimmune glial fibrillary acidic protein (GFAP) astrocytopathy is a recently defined neural autoimmune disease [1]. The common clinical features, including fever, headache, encephalopathy, involuntary movement, myelitis, and visual abnormalities, have been reported [2]. Antibodies in cerebrospinal fluid (CSF) against GFAP are biomarkers and expressed in most cases with autoimmune GFAP astrocytopathy [1]. The atypical clinical manifestations resulted in many misdiagnosis. Area postrema syndrome is included as a core clinical criterion for neuromyelitis optica spectrum disorders (NMOSD) [3] and has been rarely reported in autoimmune GFAP astrocytopathy. Herein we report a patient with autoimmune GFAP astrocytopathy whose symptom was isolated area postrema syndrome.

## Case presentation

**Fig. 1:**
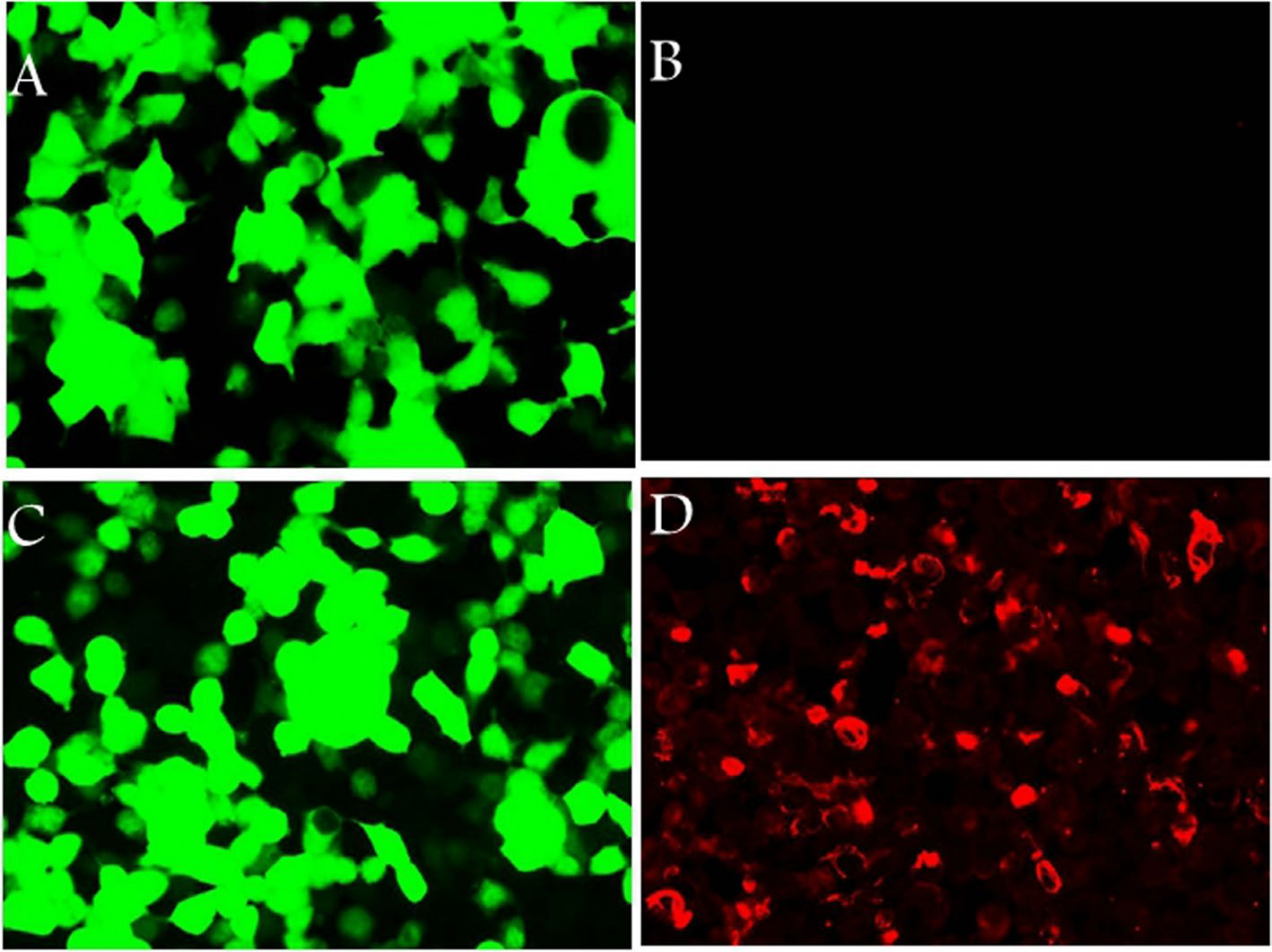
Figures of negative control cell lines. Negtive control in serum (**A** and **B**) and CSF (**C** and **D**) by transfected cell-based assay. **A** and **C** show plasmid transfection, while **B** and **D** show antigen-antibody reaction. (magnification: 200×)

**Fig. 2:**
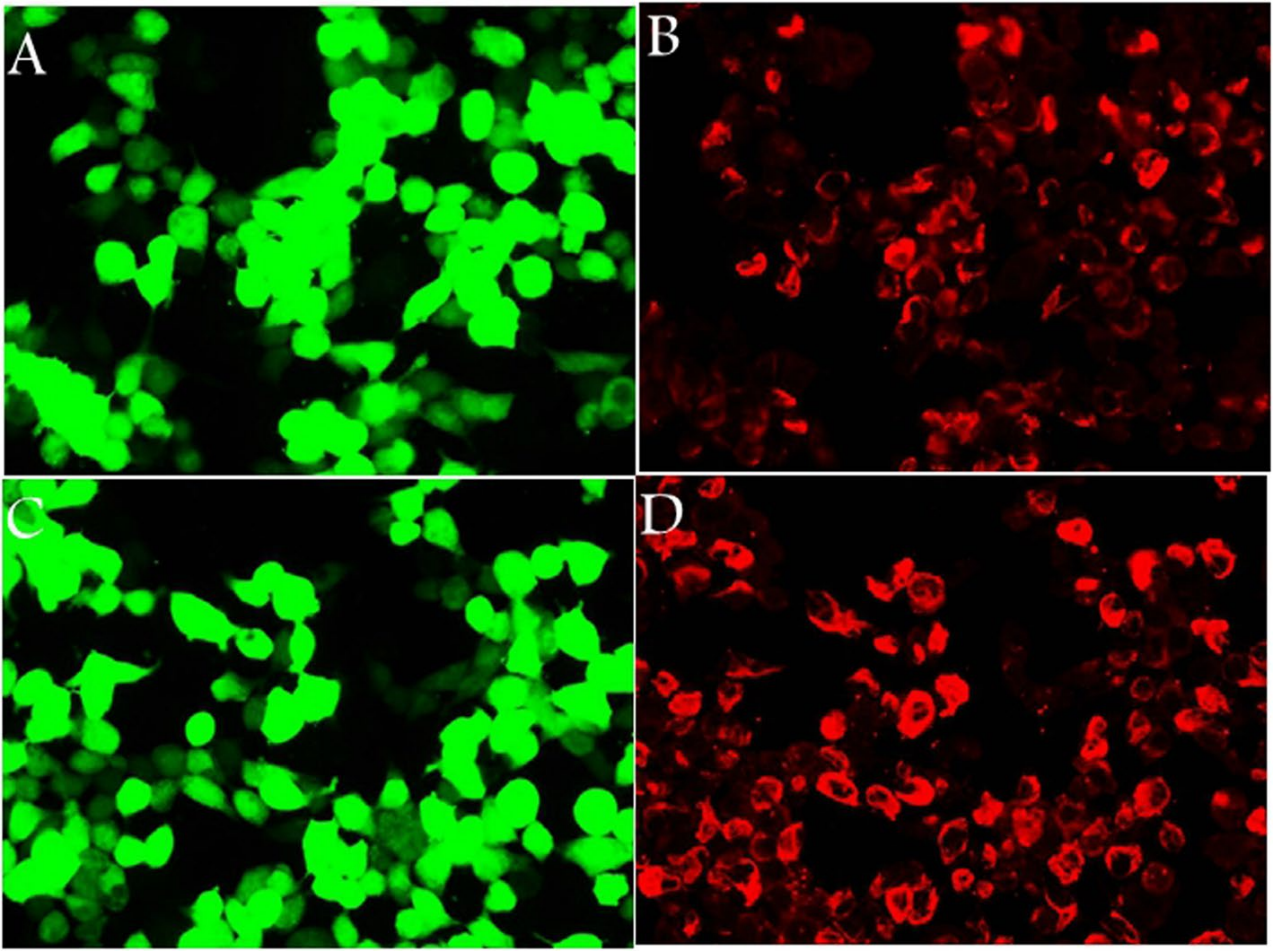
Positive GFAP-IgG in serum (B) and CSF (D) by transfected cell-based assay. **A** and **C** show plasmid transfection, while **B** and **D** show antigen-antibody reaction. (magnification: 200×)

**Fig. 3:**
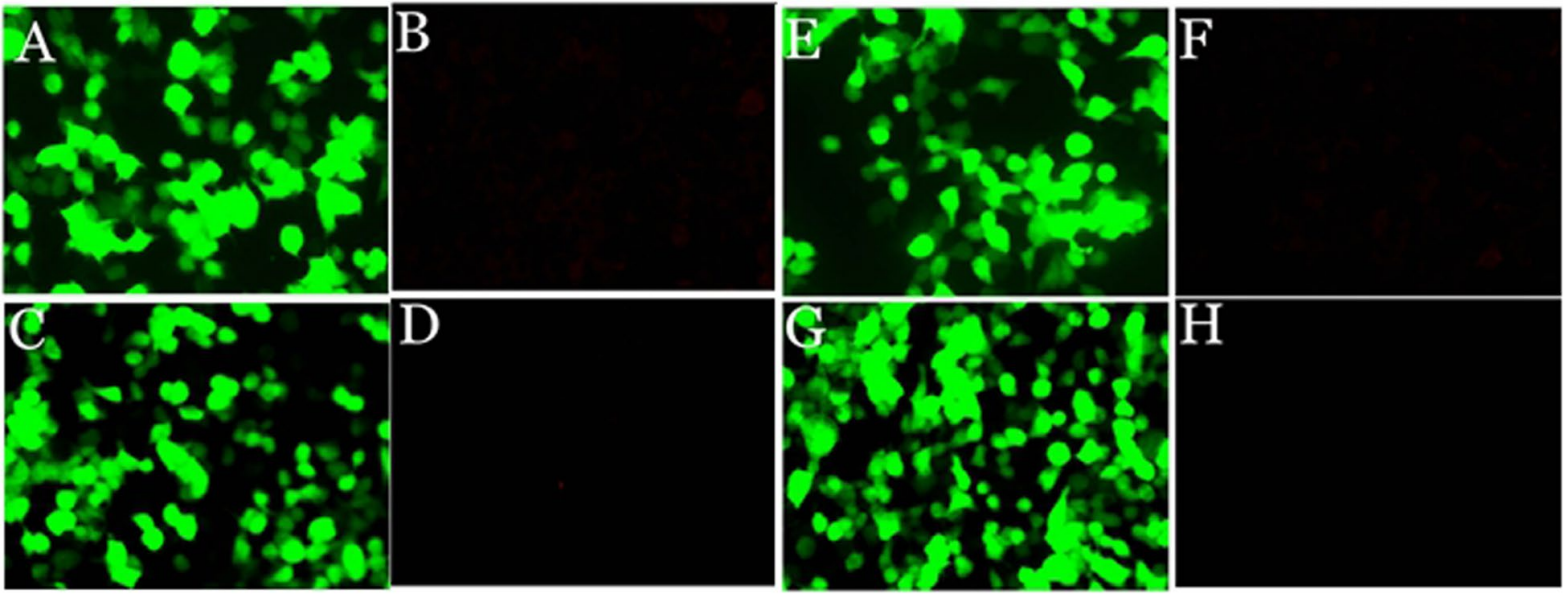
Negtive AQP4-IgG in serum (**B**) and CSF (**D**) by transfected cell-based assay. Negtive MOG-IgG in serum (**F**) and CSF (**H**) by transfected cell-based assay. **A**/**C**/**E**/**G** show plasmid transfection, while **B**/**D**/**F**/**H** show antigen-antibody reaction. (magnification: 200×)

A 61-year-old male, with a history of hypertension and cerebral infarction, was admitted to the department gastroenterology firstly with hiccup for about 1 week. Gastroscopy showed reflux esophagitis, grade C. Then he was administered digestive system protective medicines. About 2 weeks later, his symptom didn’t improve at all. He was referred to department of neurology after multidisciplinary consultation. Neurological examination was unremarkable. Chest and abdominal computed tomography (CT) was normal. Brain and cervical magnetic resonance imaging (MRI) showed no obvious abnormalities except for old cerebral infarction. No abnormal enhancement was seen. Routine blood tests for TSH, Free T4, T3, ESR, CRP were normal. Other hematologic test for autoimmune disease (IgG, IgM, IgA, Rheumatoid factor, AKA, ANA, anti-ds DNA, anti-RNP, anti-SSA/SSB, anti-Scl-70, anti-Jo-1, anti-Sm, pANCA, cANCA, anti-TPO) and tumour marks (NSE, CA 242, TPS Ag, FRS Ag, AFP, CEA, CA19–9, CA125, CA72–4, CA15–3, Cyfra 21–1) were negative. CSF or serum autoimmune encephalitis antibodies were negative: Hu, Yo, Ri, Amphiphysin, Ma2/Ta, CV2/CRMP5, MBP, NMDA, AMPA1, AMPA2, GABAB,CASPR2, LGI1, GAD65. CSF analysis showed protein 555 mg/L, white blood cells 46 × 10^6^/L (75% lymphocytes), and normal glucose level. Cell-based assays for GFAP antibodies were tested and antibodies were detected in blood and CSF. The GFAP antibody titers, compared with control (Fig. 1), in serum and CSF were 1:32 and 1:100 respectively (Fig. 2) with negative AQP4-IgG and MOG-IgG (Fig. 3). He received steroid pulse therapy (methylprednisolone, 1 g for 5 days) followed by a gradual tapering of oral prednisolone. One week after the steroid treatment, the symptom of hiccup disappeared and he could eat normally. At a follow-up appointment 3 months later, he had no relapses. He is still under follow-up.

## Discussion and conclusion

APS is defined as acute or subacute, single or combined, episodic or constant nausea, vomiting, or hiccups, persisting for at least 48 h, which cannot be attributed to any other etiology [3]. Our patient suffered hiccup at onset, and persisted for 3 weeks despite he received regular digestive system treatment. We can confirm that he had APS, although his cranial MRI didn’t show lesion in the area postrema. APS has been recognized as a characteristic presentation of NMOSD for over two decades. Thus, we examined his CSF and blood autoimmune antibodies and paid more attention to anti-AQP4, but the results demonstrated positive anti-GFAP in CSF and blood accompanying negative other antibodies including anti-AQP4 and anti-MOG. Then, the patient was diagnosed with autoimmune GFAP astrocyopathy.

Autoimmune GFAP astrocytopathy is an inflammatory central nervous system (CNS) disorder with a broad spectrum of neurological presentations. More frequent manifestations such as headache, fever, blindness, seizures or myelitis weren’t present in the case. To our knowledge, APS previously reported only in two cases with GFAP astrocyopathy [4, 5]. In addition, APS is one of the core clinical manifestations of NMOSD. Herein, we report a rare case of autoimmune GFAP astrocytopathy presented as APS. The centers for hiccup and vomiting are thought to be adjacent to the dorsal portions of the medulla oblongata, that is, area postrema (AP), an AQP4-rich region, which is often affected in patients with AQP4 antibodies [6–8]. NMOSD belongs to astrocytopathy with autoimmune GFAP astrocytopathy. Thus, it could be speculated that GFAP might be expressed in AP, which could lead to the attack of intractable hiccup on patient. Given the previous reports that autoimmune GFAP astrocytopathy with APS showed MRI lesions [4, 5], it’s probable that the patient’s mild condition leads to his negative MRI.

In all autoimmune GFAP astrocytopathy cases reported, pathological results showed extensive inflammation, particularly around small vessels [9]. We speculate that the mechanism of APS in autoimmune GFAP astrocytopathy could be inflammatory infiltration. Increased CSF lymphocyte count and protein level of our patient pointed to CNS inflammation, although he had no history of prodromal infection.

Treatment for the acute stage of autoimmune GFAP astrocytopathy includes intravenous methylprednisolon, intravenous immunoglobulin (IVIG), and plasma exchange. Long-term treatment includes oral steroids and immunosuppressants [10]. Prognosis is usually favourable. Although some studies reported that their patients had a poor response to routine treatment [11, 12], our patient responded well to steroid therapy and he hasn’t relapsed since he stopped oral prednisolone intake.

In conclusion, autoimmune GFAP astrocytopathy should be considered an etiological factor of APS. This report supports previously related case reports further and enriches the literature on spectrum of manifestation in autoimmune GFAP astrocytopathy. Whether APS is a common manifestation in autoimmune GFAP astrocytopathy, we should research further. Accurate early diagnosis and appropriate treatment are key to improve the prognosis of patients.

## Data Availability

All data generated and analyzed during this study are included in this article.

## Abbreviations

APS: Area postrema syndrome
GFAP: Autoimmune glial fibrillary acidic protein
CSF: Cerebrospinal fluid
NMOSD: Neuromyelitis optica spectrum disorders
CT: Computed tomography
MRI: Magnetic resonance imaging
CNS: Central nervous system
AP: Area postrema
IVIG: Intravenous immunoglobulin

## References

[1] Fang B, McKeon A, Hinson SR (2016). Autoimmune glial fibrillary acidic protein Astrocytopathy. A Novel Meningoencephalomyelitis. JAMA Neurol.

[2] Kunchok A, Zekeridou A, McKeon A (2019). Autoimmune glial fibrillary acidic protein astrocytopathy. Curr Opin Neurol.

[3] Wingerchuk DM, Banwell B, Bennett JL (2015). International consensus diagnostic criteria for neuromyelitis optica spectrum disorders. Neurology..

[4] Ciron J, Sourdrille F, Biotti D (2020). Area postrema syndrome: another feature of anti-GFAP encephalomyelitis. Mult Scler.

[5] Gao X, Tang Y, Yang GD (2021). Autoimmune glial fibrillary acidic protein Astrocytopathy associated with area Postrema syndrome: a case report. Front Neurol.

[6] Misu T, Fujihara K, Nakashima I (2005). Intractable hiccup and nausea with periaqueductal lesions in neuromyelitis optica. Neurology..

[7] Apiwattanakul M, Popescu BF, Matiello M (2010). Intractable vomiting as the initial presentation of neuromyelitis optica. Ann Neurol.

[8] Popescu BF, Lennon VA, Parisi JE (2011). Neuromyelitis optica unique area postrema lesions: nausea, vomiting, and pathogenic implications. Neurology..

[9] Shan F, Long Y, Qiu W (2018). Autoimmune glial fibrillary acidic protein Astrocytopathy: a review of the literature. Front Immunol.

[10] Flanagan EP, Hinson SR, Lennon VA (2017). Glial fibrillary acidic protein ImmunoglobulinG as biomarker of autoimmune Astrocytopathy: analysis of 102 patients. Ann Neurol.

[11] Yang X, Liang J (2017). Treatment of autoimmune glial fibrillary acidic protein astrocytopathy: follow-up in 7 cases. Neuroimmunomodulation..

[12] Long Y, Liang J, Xu H (2018). Autoimmune glial fibrillary acidic protein astrocytopathy in Chinese patients: a retrospective study. Eur J Neurol.

